# Long-term oncological outcomes of oncoplastic breast-conserving surgery after a 10-year follow-up – a single center experience and systematic literature review

**DOI:** 10.3389/fonc.2022.944589

**Published:** 2022-08-09

**Authors:** Jun Xian Hing, Byeong Ju Kang, Hee Jung Keum, Jeeyeon Lee, Jin Hyang Jung, Wan Wook Kim, Jung Dug Yang, Joon Seok Lee, Ho Yong Park

**Affiliations:** ^1^ Department of Surgery, School of Medicine, Kyungpook National University, Kyungpook National University Chilgok Hospital, Daegu, South Korea; ^2^ Division of Breast Surgery, Department of General Surgery, Changi General Hospital, Singapore, Singapore; ^3^ Singhealth Duke-NUS Breast Centre, Singapore Health Services Pte Ltd, Singapore, Singapore; ^4^ Department of Plastic and Reconstructive Surgery, School of Medicine, Kyungpook National University, Daegu, South Korea

**Keywords:** oncoplastic, breast-conserving surgery, oncological outcomes, volume displacement, volume replacement

## Abstract

**Aim:**

While many studies reported the oncological outcomes of oncoplastic breast-conserving surgery (OBCS), there were inherent differences in the study population, surgeons’ expertise, and classifications of techniques used. There were also limited studies with long term follow up oncological outcomes beyond 5 years. This current study aimed to compare long-term oncological outcomes of ipsilateral breast tumor recurrence (IBTR) disease-free survival (DFS) and overall survival (OS) following conventional and oncoplastic breast-conserving surgery using volume displacement and replacement techniques.

**Methods:**

Between 2009 and 2013, 539 consecutive patients who underwent breast conservation surgery including 174 oncoplastic and 376 conventional procedures were analysed. A systematic review of studies with at least five years of median follow up were performed to compare long term oncological outcomes.

**Results:**

At a median follow-up of 82.4 months, there were 23 (4.2%) locoregional recurrences, 17 (3.2%) metachronous contralateral breast cancer, 26 (4.8%) distant metastases, and 13 (2.4%) deaths. The hazard ratio of OBCS for IBTR, DFS and OS were 0.78 (95% confidence interval [CI] 0.21–2.94, p=0.78), 1.59 (95% CI, 0.88 to 2.87, p=0.12), and 2.1 (95% CI, 0.72 to 5.9, p=0.17) respectively. The 10-year IBTR-free, DFS and OS rate were 97.8%, 86.2%, and 95.7% respectively.

**Conclusion:**

There remained a dearth in well-balanced comparative studies with sufficient long-term follow-up, and our study reported long-term oncological outcomes for OBCS which were favourable of either VD or replacement techniques.

## Introduction

Historical data have shown that breast-conserving surgery followed by radiotherapy has equivalent oncological outcomes to those of mastectomy in early breast cancer (1, 2). As long-term survival after breast cancer treatment has become commonplace, more attention has been given to develop oncoplastic techniques to provide better patient and aesthetic satisfaction (3, 4). The primary role of oncoplastic breast-conserving surgery (OBCS) is to achieve oncological safety while minimizing the risk of unacceptable local deformity by allowing reconstruction of the defect and preventing the need for mastectomy (5, 6).

Following the inception of tumor-specific immediate breast reconstruction more than 20 years ago, Werner Audretsch coined the term oncoplastic surgery, and many international experts contributed to the burgeoning field of OBCS (5, 7–11). Despite the similarity in rationale behind various oncoplastic techniques, there remained differences across geographical locations in terms of surgeons’ perspectives and practices in defining OBCS (12–14). Clough described a classification based on tumor volume, location, and glandular density, while Hoffmann and Wallweiner divided breast cancer surgery into two broad types with six tiers, each of increasing complexity (13, 14). A notable consensus definition came from the American Society of Breast Surgeons, which stated that OBCS incorporated oncologic partial mastectomy with ipsilateral defect repair using volume displacement (VD) and volume replacement (VR) techniques, with contralateral symmetry surgery as appropriate (11). For small-to-moderate breast volumes, however, there was also a difference in technical considerations compared with those for larger breast volumes, which require significantly more VR techniques (6, 15–17).

Korea had been an early adopter of oncoplastic surgery but long-term follow up data remained limited. As with any surgical procedures, long-term follow up was necessary to establish safety parameters of surgical techniques. Furthermore, locoregional recurrences after breast conservation surgery could occur later than mastectomy, perhaps due to the differences in biology or presentations that led to a decision for mastectomy (18). Having previously examined the short-term oncological safety and patient-reported outcomes of various OBCS techniques (17–20), this current study aimed to compare long-term oncological safety following conventional BCS (CBCS) and OBCS, focusing on overall survival (OS), ipsilateral breast tumor recurrence (IBTR) rates, and disease-free survival (DFS). We also report the rate of positive margins (PMR) detected in intraoperative frozen sections and eventual rate of conversion to mastectomy (CMR) following BCS during a 10-year follow-up period. A literature review was performed to discuss the available data on long-term oncological outcomes with at least five years median follow up duration reported to date and how our results compared with those of other centers.

## Methods

We analyzed prospectively collected data from 539 consecutive breast cancer patients at Kyungpook National University Chilgok Hospital who underwent breast conservation surgery performed by four breast surgeons between January 2009 and December 2013. Treatment strategy was coordinated at multidisciplinary board discussions, which included breast surgeons, plastic surgeons, radiologists, pathologists, and medical and radiation oncologists. All breast conservation surgeries were performed by the breast surgeons, with oncoplastic techniques performed by either a breast or plastic surgeon.

A literature review was performed to summarise suitable studies for comparison of definitions and reported oncological outcomes (**Tables 6**, **7**) (21–40). A search was conducted through the MEDLINE database using PubMed in March 2022. Our search terms included ‘oncoplastic’ [All Fields] AND (‘breast’ [MeSH Terms] OR ‘breast’ [All Fields]) AND (‘surgery’ [Subheading] OR ‘surgery’ [All Fields] OR ‘surgical procedures, operative’ [MeSH Terms]) AND (‘oncological’ [All Fields] OR ‘outcomes’ [All Fields]). A manual search of bibliographies of relevant articles was performed.

We included single center studies reporting on various oncoplastic breast conserving surgery to ensure consistency in the reported surgical procedures. Studies with cohort size less than 50 were deemed too small; similarly, a follow up period less than 60 months inadequate to capture late recurrences and death events and hence excluded. Case series or cohort studies reporting on particular surgical techniques were also excluded as they were not generalisable to all oncoplastic breast conserving surgery. A PRISMA flowchart is available as supplementary material.

### Definition of conventional and oncoplastic BCS techniques

CBCS involved a direct skin incision, including use of a parallelogram incision overlying the index tumor to allow direct parenchymal closure. Following excision of primary breast tumors with gross margins, a frozen section of the circumferential margins was processed. The defect was closed primarily without further mobilization. When tumor cells were detected on the frozen section, more extensive resection was performed until negative frozen section results were achieved or no further surgical margins were deemed necessary. A final paraffin block of the surgical margins was examined by pathologists for the presence of tumor cells, and the presence of no stained tumor cell was defined as a negative resection margin.

OBCS was performed as described previously in detail based on general principles of oncoplastic breast surgery in small-to-moderate-sized breasts (15–17).

The procedures were divided into VD and replacement techniques. VD techniques included dual-plane glandular flap mobilization-closure, purse string suture closure of central defect, roundblock mastopexy, tennis racket incision, batwing mastopexy, rotating flap, and reduction mammoplasty (**Table 1**) (15).

**Table 1:** Oncoplastic procedures divided into volume displacement and volume replacement techniques (N=174).

Volume displacement	N=98	Volume replacement	N=76
Tennis racket	32 (18.3%)	Latissimus dorsi myocutaneous flap	23 (13.2%)
Rotating flap	31(17.8%)	Intercostal artery perforator flap	20 (11.5%)
Reduction mammoplasty	14 (8.0%)	Lateral thoracodorsal flap	18 (10.3%)
Purse string suture closure	13 (7.5%)	Thoracodorsal artery perforator flap	11 (6.3%)
Batwing mastopexy	4 (2.3%)	Thoracoepigastric flap	2 (1.1%)
Glandular flap	4 (2.3%)	Adipofascial flap	2 (1.1%)

In cases of anticipated significant breast volume loss, VR techniques were individualized according to the excised breast volume and tumor location with planned use of either adipofascial flap, lateral thoracodorsal flap (LTD), intercostal artery perforators (ICAP), thoracodorsal artery perforator (TDAP), thoracoepigastric (TE), or latissimus dorsi (LD). LD myocutaneous flaps were preferred for excised specimen >150 g (16, 17).

### Patient and tumor characteristics

Patient demographics, surgical details, clinicopathological characteristics, including clinical tumor size, specimen weight, tumor type, pathological tumor size, pathological tumor, nodal stage, receptor status, grade, presence of neoadjuvant and adjuvant therapy, metachronous contralateral breast cancer, locoregional and distant disease recurrences, and death were recorded (**Table 2**).

**Table 2:** Baseline characteristics of patients who underwent conventional and oncoplastic breast-conserving surgery (BCS).

	All, N=539	Conventional BCS, N=365	Oncoplastic BCS, N=174	p-value	
Mean age (years, ± SD)	49.4 ± 9.0	50.7 ± 9.2	46.5 ± 7.5	<0.001	
Mean body mass index (kg/m^2^ ± SD)	23.6 ± 3.3	23.6 ± 3.5	23.5 ± 3.1	0.55
Mean specimen weight ± SD, g	68.1 ± 46.6	53.1 ± 26.8	96.3 ± 60.9	<0.001
Mean clinical tumor size (cm ± SD)	1.7 0.8	1.5 ± 0.7	2.1 ± 1.1	<0.001
Tumor location by quadrant (n, %)*				
Central Upper outer quadrant Upper inner quadrant Lower inner quadrant Lower outer quadrant Multifocal	34 140 61 16 32 12	9 (6.0%) 87 (58.3%) 36 (24.2%) 5 (3.3%) 10 (6.7) 2 (1.3%)	26 (17.4%) 53 (35.6%) 25 (16.8%) 11 (7.4%) 22 (14.8%) 10 (6.7%)	<0.001
Mean pathological tumor size (cm ± SD)	1.5 ± 0.7	1.3 ± 0.6	1.8 ± 0.8	<0.001
Axillary lymph node dissection	108 (20.5%)	66 (18.5%)	42 (24.6%)	0.10
Tumor type				
DCIS/pleomorphic LCIS Invasive ductal carcinoma Invasive lobular carcinoma Mixed/others	26 (4.8%) 469 (87.0%) 17 (3.1%) 27 (5.0%)	19 (5.2%) 318 (87.1%) 10 (2.7%) 18 (4.9%)	7 (4.0%) 151 (86.8%) 7 (4.0%) 9 (5.2%)	0.81
Pathological tumour staging				
0 1 2 3	23 (4.3%) 401 (74.5%) 113 (21.0%) 1 (0.2%)	16 (4.3%) 295 (80.1%) 54 (14.8%) 0	7 (4.0%) 106 (60.9%) 59 (33.9%) 1 (0.5%)	<0.001
Pathological nodal staging				
0 1 2 3	430 (80.0%) 89 (16.5%) 14 (2.6%) 5 (0.9%)	299 (81.9%) 55 (15.1%) 6 (1.6%) 5 (1.4%)	131 (75.3%) 34 (19.5%) 8 (4.6%) 0	0.03
Pathological TNM stage				
0 1 2 3	36 (6.1%) 325 (60.3%) 158 (29.3%) 20 (3.71%)	23 (6.0%) 240 (65.7%) 91 (25.2%) 11 (3.0%)	13 (7.5%) 85 (48.9%) 67 (38.5%) 9 (5.2%)	0.002
Receptor profile				
HR+ Her2- HR+ Her2+ HR- Her2- HR- Her2+	362 (67.2%) 57 (10.6%) 86 (16.0%) 25 (4.6%)	249 (68.5%) 38 (10.4%) 57 (15.6%) 15 (4.1%)	113 (64.9%) 19 (10.9%) 29 (16.7%) 10 (5.7%)	0.89
Grade				
1 2 3	114 (22.8%) 268 (53.5%) 115 (23.0%)	86 (23.5%) 184 (50.4%) 67 (18.3%)	28 (16.1%) 84 (48.3%) 48 (27.6%)	0.04
Positive frozen margin status	36 (6.8%)	32 (9.0%)	4 (2.3%)	0.04
Neoadjuvant therapy	19 (3.5%)	12(3.2%)	7 (4.0%)	0.67
Adjuvant chemotherapy	308 (57.1%)	196 (53.7%)	112(64.7%)	0.02
Adjuvant radiotherapy	467 (86.6%)	325 (89.0%)	142 (81.6%)	0.02
Adjuvant hormonal therapy	407 (75.5%)	282 (77.3%)	125 (71.8%)	0.17
Contralateral breast cancer	17 (3.2%)	10 (2.7%)	7 (4.0%)	0.42
Ipsilateral breast tumor recurrence	11 (2.0%)	8 (2.1%)	3 (1.7%)	0.78
Locoregional recurrence	23 (4.2%)	14 (3.8%)	9 (5.7%)	0.47
Distant recurrence	26 (4.8%)	16 (4.8%)	10(5.7%)	0.49
Death	13 (2.4%)	7 (1.9%)	6 (3.5%)	0.28

SD = standard deviation; DCIS = ductal carcinoma in situ; LCIS: lobular carcinoma in situ; HR = Hormone receptor (estrogen receptor and progesterone receptor); Her2 = human epidermal growth factor receptor 2.

*Available data from 149 consecutive conventional breast conserving surgery was compared with 147 oncoplastic breast conserving surgery between 2011 and 2013.

### Follow-up

Patients were followed up after surgery using a standardized protocol. After completing adjuvant treatments, frequency of follow-up was biannually for the first 2 years and annually for 5 to 10 years. Locoregional recurrence or distant metastasis was evaluated with clinical examination, blood tests including tumor markers, mammography, breast ultrasonography, with or without magnetic resonance imaging, bone scans, and positron emission tomography/computed tomography.

### Oncological outcomes

The oncological outcomes assessed include OS, DFS with disease events defined as local or regional recurrences, distant recurrences, and metachronous contralateral breast cancer.

### Statistical analysis

Statistical analysis was performed in March 2022 using Stata software, v17.0 (StataCorp); a statistically significant difference was concluded when p<0.05. Categorical variables were analyzed using the chi-square or Fisher exact test. Continuous variables were analyzed using the Student’s t-test. The Kaplan–Meier method was used to estimate survival function, and the log-rank test was used to compare survival functions. The univariate Cox proportional hazard regression model was also used to examine the correlation of clinically relevant covariates that were likely to affect oncological outcomes. These included patient age, tumor grade, hormonal profile, pathological tumor stage, nodal disease, and adjuvant therapy received. A multivariate analysis was performed with variables with significant p-values in the univariate model.

The study was approved by the Institutional Review Board of Kyungpook National University (2015-05-205) and conducted in compliance with the principles of the Declaration of Helsinki.

## Results

Of the 539 patients who were analyzed, 365 (67.7%) patients underwent CBCS while 174 (32.3%) underwent OBCS. Of the 174 cases of OBCS, VR techniques were utilized in 98 (56.3%) cases, while VD techniques were utilized in 76 (43.7%) cases. **Table 1** shows the breakdown of oncoplastic procedures in descending order. The most commonly employed techniques among the oncoplastic procedures were tennis racket incision (32), rotating flap (31), and LD myocutaneous flap (23). Five patients who defaulted further clinical visits or transferred care to other hospitals were considered lost to follow up.

### Patient characteristics

Patients who underwent CBCS were older (50.7 vs. 46.5 years old), had smaller clinical tumor size (1.5 cm vs. 2.1cm), smaller specimen weight (53.1 g vs. 96.3 g), and smaller pathological tumor size (1.3 cm vs. 1.8 cm) compared to those who underwent OBCS. In terms of tumor characteristics, patients who underwent CBCS had earlier pathological T and N stage compared to those who underwent OBCS, while there was no statistically significant difference in histology subtype, grade, or hormone profile (**Table 2**) among the two groups.

### Tumor location

OBCS was performed on a higher proportion of central (17.4%), lower outer quadrant (14.8%), lower inner quadrant (7.4%), and multifocal tumors (6.7%) than CBCS. The majority of all CBCS was performed on upper outer quadrant tumors (58.5%).

### Intraoperatively detected involved margins on frozen section

The rate of intraoperatively detected involved margins on frozen section was higher in the CBCS than in the oncoplastic group, and further margins were excised intraoperatively. Three patients required completion mastectomy for close or involved final margins.

### Disease recurrence, overall survival, and success of breast conservation surgery at 10 years

At a median follow-up of 82.4 months, (range, 1.4–156.7 months) there were 23 (4.2%) locoregional recurrences of which 11 had ipsilateral breast tumor recurrences, 17 (3.2%) metachronous contralateral breast cancer, 26 (4.8%) distant metastases, and 13 (2.4%) deaths. The hazard ratio of OBCS for IBTR, DFS and OS were 0.78 (95% confidence interval [CI] 0.21–2.94, p=0.78), 1.59 (95% CI, 0.88 to 2.87, p=0.12), and 2.1 (95% CI, 0.72 to 5.9, p=0.17) respectively. The 10-year IBTR-free, DFS and OS rate were 97.8%, 86.2%, and 95.7% respectively. Overall, five patients underwent mastectomy either from involved margins or disease recurrence, giving a successful BCS rate of 99.1%.

### Statistical analysis of oncological outcomes

**Figure 1:**
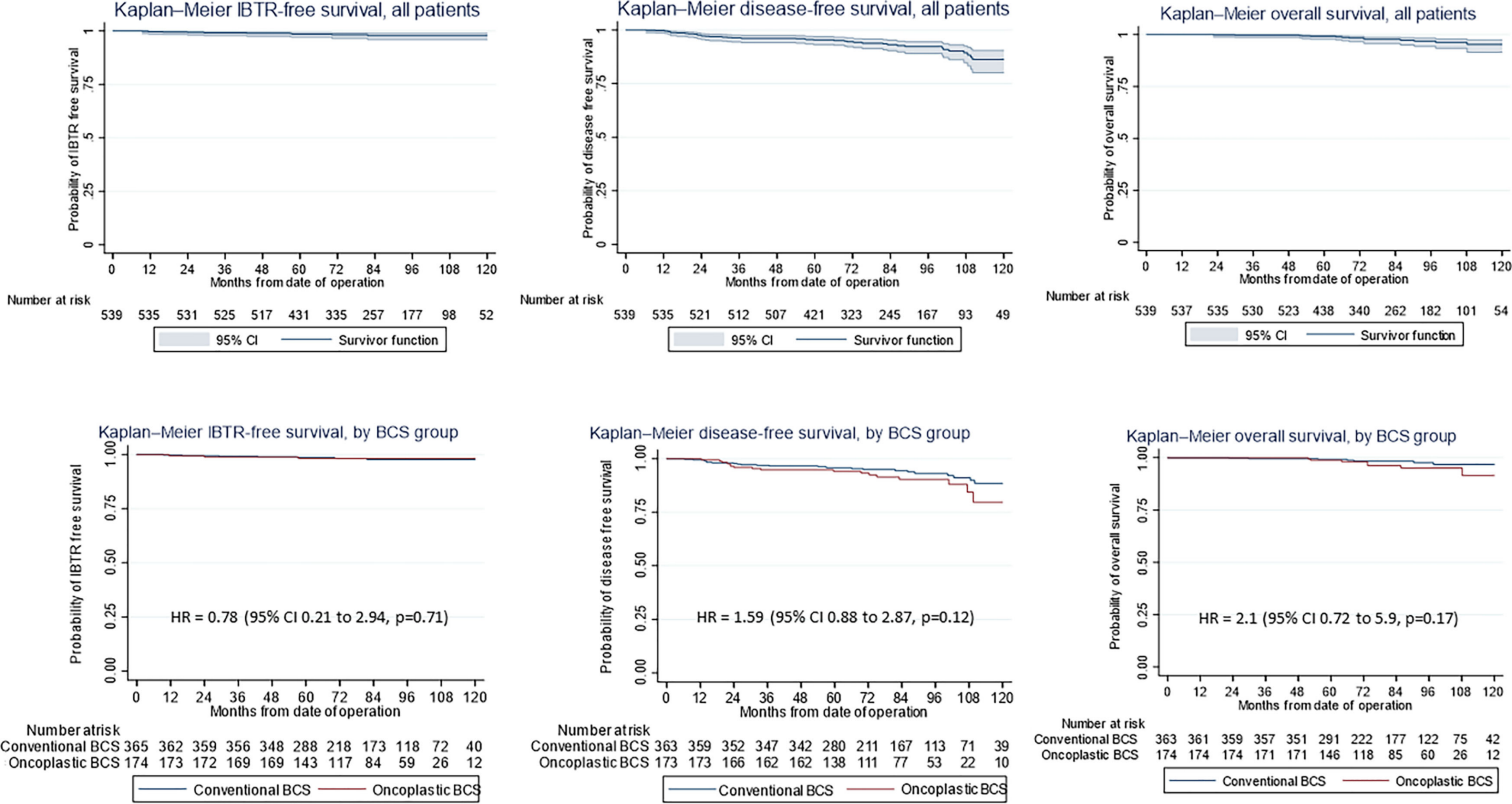
(First row) Kaplan–Meier estimates of (Left) ipsilateral breast tumor recurrence (IBTR)-free survival, (Middle) disease-free survival (DFS), and (Right) overall survival (OS) curves (shown with 95% confidence level) for all patients undergoing breast-conserving surgery (BCS) and (second row) by conventional (CBCS) versus oncoplastic breast-conserving surgery (OBCS) group.

**Figure 2:**
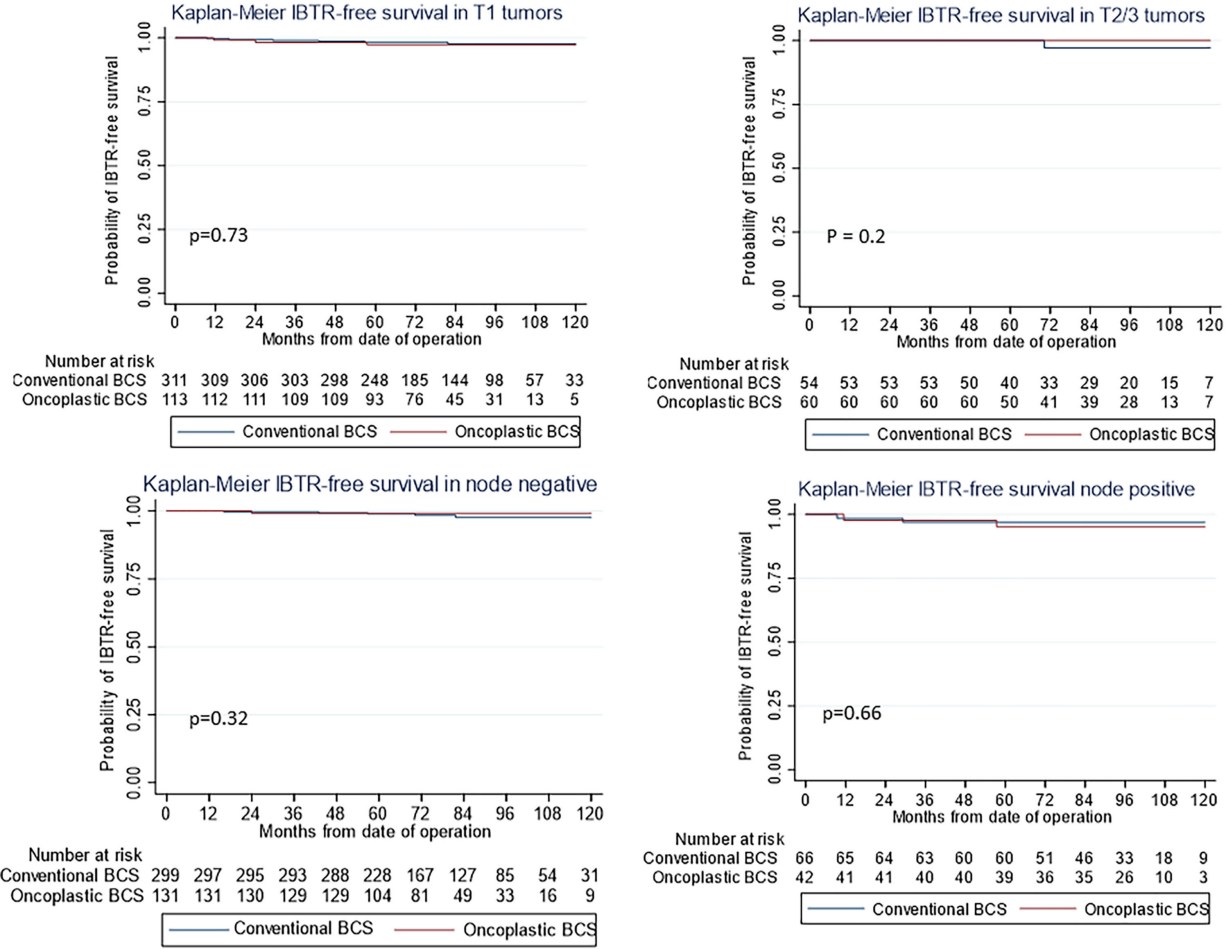
Kaplan–Meier estimates for ipsilateral breast tumor recurrence (IBTR)-free survival by (First Row) pathological tumor stage (first row) and (Second Row) nodal stage (second row) showing no difference between oncoplastic and conventional breast-conserving surgery (BCS).

**Figure 3:**
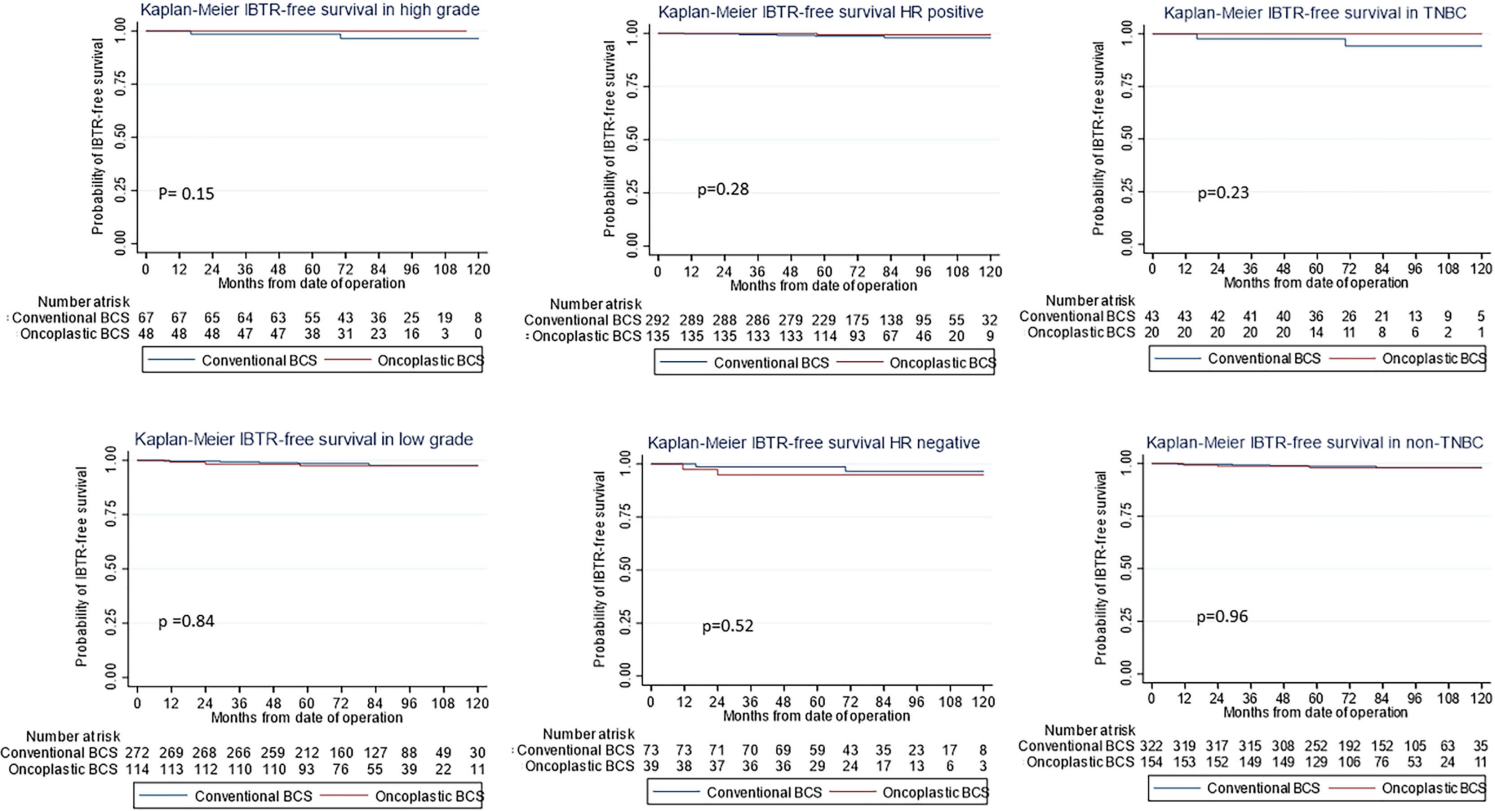
Kaplan–Meier estimates for ipsilateral breast tumor recurrence (IBTR)-free survival stratified by (Left) high-grade, (Middle) hormone-positive tumors, and (Right) triple negative breast cancer subtypes (first row) and others (second row) showing no difference between oncoplastic and conventional breast-conserving surgery (BCS).

The use of oncoplastic surgery was not associated with a higher likelihood of IBTR or death in the Cox regression model analysis (**Tables 3**, **4**, **5**). Patients who underwent adjuvant chemotherapy had significantly lower IBTR rates, with a hazard ratio of 0.25 (95% CI, 0.07 to 0.98). Regarding OS, higher histological grade was significantly associated with higher risk of death, with a hazard ratio of 9.56 (95% CI, 2.41 to 37.86) (**Tables 3** and **5**). Univariate analysis was performed using the log-rank method stratified by tumor histological grade, pathological tumor staging, nodal disease, and hormone receptor profile. There was no difference in IBTR-free survival when performing OBCS after stratifying by high-grade tumors; larger tumors (T2/3); and node positive, hormone receptor-positive, or triple negative breast tumors (**Figures 1**, **2**, **3**).

**Table 3:** Univariate and multivariate Cox regression analysis with ipsilateral breast tumor recurrence-free survival as an endpoint.

	No. of cases, N=539	No. of IBTR, N=11	Univariate HR	p	Multivariate HR*	p
Type of BCS						
Oncoplastic Conventional	174 365	3 8	0.78 (0.21–2.94) Ref	0.71	0.89 (0.23-3.39) Ref	0.87
ALND						
Yes No Missing	108 420 11	5 6	2.91 (0.88–9.63) Ref	0.09	–	–
Age	539	11	1.0 (0.96–1.08)	0.55	–	–
Histological subtype						
IDC Others	469 70	11 0	Ref 0.67 (0.17–2.68)	0.55	–	–
Grade						
Grade 1/2 Grade 3 Missing	386 115 38	9 2	Ref 0.77 (0.17–3.60)	0.74	–	–
Tumor stage						
T1 T2/3	424 115	10 1	Ref 0.34 (0.04–2.69)	0.24	–	–
Nodal stage						
Node negative Node positive	430 108	7 4	Ref 2.06 (0.60–7.08)	0.27	–	–
Hormone receptor						
Positive Negative Triple negative Yes No	427 111 63 476	7 4 2 9	0.44 (0.21–1.51) Ref 1.75 (0.37–8.10) Ref	0.21 0.50	- -	–
Adjuvant chemotherapy						
Yes No	308 231	8 3	0.26 (0.07–0.96) Ref	0.03	0.25 (0.07–0.98) Ref	0.047
Adjuvant radiotherapy						
Yes No	467 72	9 2	0.71 (0.15–3.28) Ref	0.67	–	–
Adjuvant hormonal therapy						
Yes No	407 132	7 4	0.52 (0.15–1.82) Ref	0.32	–	–

*Variables with p-values <0.05 in the univariate analysis were included in the multivariate analysis.

**Table 4:** Univariate and multivariate Cox regression analysis with disease-free survival^+^ as an endpoint.

	No. of cases, N=539	No. of recurrences, N=47	Univariate HR	p	Multivariate HR*	p
Type of BCS						
Oncoplastic Conventional	174 365	19 28	1.59 (0.88–2.88) Ref	0.13	1.95 (1.04–3.64) Ref	0.04
ALND						
Yes No Missing	108 420 11	16 31	1.79 (0.97–3.31) Ref	0.07	–	–
Age	539	47	1.02 (1.00–1.05)	0.05	1.05 (1.00–1.07)	0.03
Histological subtype						
IDC Others	469 70	43 4	Ref 2.23 (0.31–16.2)	0.48	–	–
Grade						
Grade 1/2 Grade 3 Missing	386 115 38	30 17	2.15 (1.17–3.94)	0.02	1.80 (0.91–3.55)	0.09
Tumor stage						
T1 T2/3	423 115	35 12	1.21 (0.62–2.34)	0.57	–	–
Nodal stage						
Node negative Node positive	430 108	33 14	1.47 (0.78–2.76) Ref	0.24	–	–
Hormone receptor						
Positive Negative Triple negative Yes No	427 111 63 476	31 16 6 41	0.49 (0.27–0.91) Ref 1.13 (0.48–2.68) Ref	0.03 0.78	0.70 (0.35–1.39) -	0.31 -
Adjuvant chemotherapy						
Yes No	308 231	26 21	0.84 (0.47–1.51) Ref	0.57	–	–
Adjuvant radiotherapy						
Yes No	467 72	37 10	0.62 (0.31–1.25) Ref	0.20	–	–
Adjuvant hormonal therapy						
Yes No	407 132	31 16	0.57 (0.31–1.03) Ref	0.07	–	–

^+^Disease-free survival events were defined as any ipsilateral or contralateral breast recurrence (invasive or non-invasive) or regional or distant metastases.

*Variables with p-values <0.05 in the univariate analysis were included in a multivariate analysis.

HR = hazard ratio; Ref = Reference; BCS = breast conservation surgery; ALND = axillary lymph node dissection; IDC = invasive ductal carcinoma.

**Table 5:** Univariate and multivariate Cox regression analysis with overall survival as an endpoint.

	No. of cases,N=539	No. of deaths, N=13	Univariable HR	p	Multivariate HR*	p
Type of BCS						
Oncoplastic Conventional	174 365	6 7	1.78 (0.60–5.29) Ref	0.31	1.82 (0.55–5.97)	0.33
Age	539		2.61 (0.87–7.82)	0.10	–	–
Histological subtype						
IDC Others	469 70	13 0	1.03 (0.98–1.09) Ref	0.29	–	–
Grade						
Grade 1/2 Grade 3 Missing	386 115 38	3 10	Ref 1.78 (2.96–39.2)	0.0001	Ref 9.56 (2.41–37.86)	0.001
Tumor stage						
T1 T2/3 Missing	423 115 1	10 3	Ref 1.01 (0.28–3.68)	0.98	–	–
Nodal stage						
Node negative Node positive Missing	430 108 1	9 4	Ref 1.35 (0.41–4.42)	0.62	–	–
Hormone receptor						
Positive Negative Triple negative Yes No	427 111 63 476	7 6 3 10	0.31 (0.10–0.91) Ref 2.32 (0.63–8.43) Ref	0.04 0.24	0.80 (0.25–2.56) Ref -	0.70 -
Adjuvant chemotherapy						
Yes No	308 231	10 3	2.21 (0.61–8.06) Ref	0.20	–	–
Adjuvant radiotherapy						
Yes No	467 72	11 2	0.92 (0.20-4.14) Ref	0.91	–	–
Adjuvant hormonal therapy						
Yes No	407 132	7 6	0.36 (0.12–1.08) Ref	0.08	–	–

*Variables with p-values <0.05 in the univariate analysis were included in the multivariate analysis.

HR = hazard ratio; Ref = Reference; BCS = breast conservation surgery; ALND = axillary lymph node dissection; IDC = invasive ductal carcinoma.

Comparison of our current study with other similar studies reporting long-term oncological outcomes are summarized in **Tables 6** and **7**.

**Table 6:** Retrospective studies showing single center studies with large cohort and long term follow up, comparing definitions of OBCS and breakdown of oncoplastic procedures by year of published study.

Study and center	Year	Cohort size	Classification of OBCS	Percentage of VR among OBCS
Our study, Kyungpook National University Chilgok Hospital, Korea	2022	539 -174 (OBCS) -365 (CBCS)	VD and VR	43.7
Oh, Seoul National University Hospital, Korea ^28^	2021	742 -371 (OBCS) -371 (CBCS)	VD and VR	5.4
Kelemen, National Institute of Oncology, Hungary ^29^	2019	756 -378 (OBCS) -378 (CBCS)	Clough bilevel	Excluded VR
Calabrese, Sapienza University Italy ^36^	2018	1024 (All OBCS)	VD	Excluded VR
Clough, Paris Breast Centre, France ^30^	2017	350 (All OBCS)	Clough Bilevel	Excluded VR
Mansell, Victoria & Western Infirmary, UK ^31^	2017	666 -108 (OBCS) -558 (CBCS)	Clough Bilevel	13.5
De Lorenzi, European Institute of Oncology, Italy 32	2016	1362 -454 (OBCS) -908 (CBCS)	Tumor location Includes VD, VR and implant	10.3
Chakravorty, Royal Marsden, UK ^34^	2012	590 -150 (OBCS) -440 (CBCS)	By location and 3 standardized VD	Excluded VR
Fitoussi, Institut Curie Paris, France ^35^	2010	540 (All OBCS)	Tumor location Aesthetic vs combination	Excluded VR

*No available data on breakdown

VD = volume displacement; VR = volume replacement; OBCS = oncoplastic breast-conserving surgery; CBCS = conventional breast-conserving surgery.

**Table 7:** Retrospective studies showing oncologic outcomes of oncoplastic breast conservation surgery according to surgeons, operation period, and follow-up interval to show directly reported results for local recurrence, disease-free survival, and overall survival.

Study	Surgeons	Operation period	Follow-up, months	IBTR rates (%)	Disease-free survival, %	Overall survival, %
Our study	Both breast and plastic surgeons	5 years (2009–2013)	82.5 (all) 82.9 (OBCS) 81.4 (CBCS)	2.2 (10 years, all) 1.8 (10 years OBCS) 2.4 (10 years CBCS)	86.2 (10 years, all) 79.7 (10 years, OBCS) 88.5 (10 years, CBCS)	95.7 (10 years, all) 92.6 (10 years, OBCS) 96.8 10 years, CBCS)
Oh ^28^	Not specified	4 years (2011–2014)	84.4 (OBCS) 87.9 (CBCS)	3.1 (5 years, OBCS) 1.4 (5 years, CBCS)	92.9 (5 years, OBCS) 94.5 (5 years, CBCS)	–
Kelemen ^29^	2 breast surgeons	7 years (2010–2017)	51 (OBCS) 52 (CBCS)	–	88.5 (5 years, OBCS) 78.2 (5 years, CBCS)	100 (5 years, OBCS) 97.3 (5 years CBCS)
Calabrese ^36^	Breast and plastic surgeons	11 years (2000-2010)	74.2 (all)	4.7 (all)	95.0 (all)	98.4
Clough ^30^	Not specified	13 years (2004–2016)	55 (all)	–	84.8	95.1 (5 years)
Mansell ^31^	Either breast or plastic surgeons	4 years (2009–2012)	56.2 (all) 56.8 (OBCS) 57.2 (CBCS)	2 (5 years, OBCS) 3.4 (5 years, CBCS)	90.7 (5 years, OBCS) 93.2 (5 years, CBCS)	98.1 (5 years, OBCS) 95.1 (5 years, CBCS)
De Lorenzi ^32^	Not specified	9 years (2000–2008)	86.4	6.7 (10 years, OBCS) 4.2 (10 years, CBCS)	69 (10 years, OBCS) 73.1 (10 years, CBCS)	91.4 (10 years, OBCS) 91.3 (10 years, CBCS)
Chakravorty ^34^	2 oncoplastic surgeons	7 years (2003–2010)	28	4.3 (Projected 6 years, OBCS) 3.7 (Projected 6 years, CBCS)	–	–
Fitoussi ^35^	Not specified	22 years (1986–2008)	49	6.8 (5 years, all)	87.9 (5 years, all)	92.9 (5 years, all)

OBCS = oncoplastic breast-conserving surgery; CBCS = conventional breast-conserving surgery.

## Discussion

Over the last two decades, oncoplastic breast surgery quickly gained widespread acceptance as a standard of care option that balanced oncological and aesthetic outcomes of oncological resection in breast cancer management (21–27). The main findings of this study were that there was an overall low rate of IBTR (2.2%) and death (4.3%) observed in this cohort of 539 patients after a median follow-up of 82.4 months. This study had one of the largest single center cohorts with a long follow-up period (**Table 7**). Like other studies, IBTR rates were estimated to be between 1.4% and 14.6%, and 10-year OS rates were approximately 90.2–100%. Stratified analysis did not reveal any associated difference in survival outcomes in larger tumors, higher grade disease, or disease with a nodal burden. The observed outcomes could be the result of other factors, such as younger age (mean age <50 year), earlier disease stage (majority stage 1 and 2), favorable histological subtype, and generally high uptake rates of adjuvant therapies such as chemotherapy, radiotherapy, and hormonal therapy, when indicated. The proportion of cases with neoadjuvant chemotherapy was lower than expected in current practice; this might be because of the trend of favoring upfront surgery 10 years ago. However, as this was a retrospective cohort analysis, the cumulative incidence of events could also be underestimated because of a loss to follow-up or selection bias.

We also observed a similar trend that oncoplastic techniques allowed higher resection volumes for larger tumors and reduced intraoperative positive margin rates. Large systematic reviews showed that oncoplastic surgery was more frequently performed in younger patients who required greater breast volume removal for larger tumors (23, 25, 27). While this may not translate to any survival benefit, there could be improvement in patients’ satisfaction rates given the lower rate of reoperation and conversion to mastectomy (28).

Our literature review showed that there were several registry studies and meta-analyses published on oncological outcomes of oncoplastic breast surgery (21–42). However, we must caution that conclusions drawn from such meta-analyses or registries have inherent limitations. Many studies have difficulties pooling study subjects together due to the heterogeneity of the study population, surgeons’ expertise, and techniques and classifications used (21–26, 42). Therefore, we analyzed the different definitions and breakdowns of oncoplastic techniques used across various studies (**Table 5**). We noted that majority of the studies were small observational studies on specific techniques, limiting their generalizability and had to be excluded from the meta-analysis. Most had a limited cohort size or a barely sufficient follow-up duration to fully capture recurrence or death events. As it would be impossible to conduct any randomized control trial studying conventional and oncoplastic techniques because of ethical considerations, large cohort studies with long-term follow-up could be regarded as the highest level of evidence.

This study generated fresh data on long-term outcomes so as to compare with the reported standards over the last decade. First, the main strength of this study was the clear definition of procedures performed with balanced representations of both VD and replacement techniques. Second, consistency in surgical standards was maintained in the procedures performed by a dedicated oncoplastic team made up of both breast and plastic surgeons. Third, these 539 patients were followed up for more than a median of 80 months to allow for more valid capture of long-term outcomes.

Next, we examined the most commonly used definition of the Clough classification in the literature. The Clough classification of oncoplastic techniques primarily considers the excision volume ratio, requirement of skin excision for reshaping or mammoplasty, and tumor location. However, VR techniques were notably excluded because of their primary use in smaller breasts (13). Similarly, we found that many comparative studies with long-term outcomes reported a disproportionately low number of VD techniques, mainly level 2 oncoplastic mammoplasty with little or no representation of VR techniques. In our and many other East Asian populations, we adopted similar principles of deciding the type of oncoplastic procedures such as breast-to-tumor volume ratio, and tumor locations, but it was also proposed that an absolute value of tumor volume excised in itself could be an indication for VR techniques in small-to-moderate-sized breasts. These may be due to inherent differences in the patients’ morphometric characteristics or influenced by different cultural beliefs and resource settings (15, 41). In a smaller native breast with less space for VD maneuvers, a different threshold for VR techniques may apply. Evidence also shows that patients are more accepting of VR options and have good functional outcomes regardless of the VR technique (17).

As a result, our percentage of VR performed among OBCS was the highest among the selected studies, with VR techniques accounting for 43% of all oncoplastic procedures. Most of the other studies either had less than 10% of procedures represented by VR, or did not specify the type of reconstruction techniques at all. Our cohort also showed that the LD myocutaneous flap was the most commonly used VR techniques followed by chest wall perforator flaps. This was concordant to our finding that the LD flap was the largest and the most commonly reported VR technique as a single cohort series in the literature (20, 25, 37–42). However, we did not report and compare the oncological results from these studies that only focused on singular technique such as LD flap or omental flap reconstruction because they would have limited generalizability to other oncoplastic techniques and patient selection (40, 41).

We maintained that both VD and VR techniques formed the fundamentals of oncoplastic techniques and would not need to be separately studied from each other. Hence, it remained vital to establish comparable oncological outcomes of various oncoplastic techniques to reassure patients that oncoplastic breast surgery would not compromise on oncological safety in the long run, and that both aesthetic outcomes and patient satisfaction were equally important performance indicators in the treatment of breast cancer.

### Limitations

The main limitations of the study were largely in its retrospective nature, which could lead to underestimated incidence rates due to the nature of selection bias and loss to follow-up. The surgical teams involved a dedicated oncoplastic team including both breast and plastic surgeons; consequently, these findings may not be logistically reproducible in all centers. We acknowledged that there were many confounding factors that could affect oncological outcomes and tried to address these by adjusting for the variables in the statistical analysis. However, considering the limitations of cohort size and event rates, it would be prudent to avoid generating too many hypotheses regarding secondary analysis findings but rather appreciate the general theme of oncological safety established across various tumor characteristics and adjuvant therapies provided in our study population. We also noted there was a low percentage of patients treated with neoadjuvant chemotherapy in our cohort. Neoadjuvant chemotherapy has gained much traction in its role in increasing rates of breast conservation; therefore, future research should be directed to study its influence on long-term oncological outcomes (43).

## Conclusion

Our review of existing literature on the oncological outcomes of OBCS highlighted the dearth in well-balanced comparative studies with sufficient long-term follow-up, and reported our center’s own long-term oncological outcomes for OBCS to support the use of either VD or replacement techniques.

## Data availability statement

The raw data supporting the conclusions of this article will be made available by the authors, without undue reservation.

## Ethics statement

The studies involving human participants were reviewed and approved by Kyungpook National University Chilgok Hospital. Written informed consent for participation was not required for this study in accordance with the national legislation and the institutional requirements.

## Acknowledgments

We would like to thank Editage (www.editage.co.kr) for English language editing.

## Author contributions

All authors listed have made a substantial, direct, and intellectual contribution to the work and approved it for publication.

## Conflict of interest

The authors declare that the research was conducted in the absence of any commercial or financial relationships that could be construed as a potential conflict of interest.

## Publisher’s note

All claims expressed in this article are solely those of the authors and do not necessarily represent those of their affiliated organizations, or those of the publisher, the editors and the reviewers. Any product that may be evaluated in this article, or claim that may be made by its manufacturer, is not guaranteed or endorsed by the publisher.
