# Supplementary material for: Long-term oncological outcomes of oncoplastic breast-conserving surgery after a 10-year follow-up – a single center experience and systematic literature review
Source: Front Oncol. 2022 Aug 9;12:944589. doi: 10.3389/fonc.2022.944589 (PMC9396304; doi:10.3389/fonc.2022.944589)
Supplement: Supplementary file 1 [file DataSheet_1.docx]

Supplementary data on PRISMA flowchart of literature review

Records removed *before screening* due to:

- irrelevant titles
- (n = 226)

Records identified from Pubmed

(n = 286)

**Identification**

Records excluded due to

- Review articles
- Case reports/series
- Did not report oncological outcomes and no full text available

(n = 45)

Records screened using title and abstract

(n = 60)

**Screening**

Records excluded due to

- Insufficient oncological outcomes for direct comparison
- Insufficient cohort size <50
- Insufficient long term followup <60months

(n=7)

Bibliographic references searched from 7 systematic reviews ^20-26^

Full text articles assessed for eligibility and bibliographic reference search

(n = 15) ^27-41^

Studies presented in summary table

(n = 8)

**Presented**
